# Integrating genome and transcriptome-wide data to explore the expression dynamics of *TCP* genes in *Pisum sativum* under salt stress

**DOI:** 10.3389/fpls.2025.1580890

**Published:** 2025-05-01

**Authors:** Song Fangyuan, Li Yong, Jin Huang, Guo Zhiyue, Deng Wen

**Affiliations:** Cash Crops Research Institute, Hubei Academy of Agricultural Sciences, Wuhan, China

**Keywords:** TCP gene family, *Pisum sativum*, salt stress, tissue-specific expression, WGCNA

## Abstract

Salt stress severely restricts plant growth and productivity. TCP genes, which are plant-specific transcription factors, play a crucial role in the stress response. However, their functions in pea (*Pisum sativum*) remain poorly understood. Here, we identified 21 *PsTCP* genes in pea, classified into Class I (PCF) and Class II (CYC/TB1 and CIN) through phylogenetic analysis. While physicochemical properties varied significantly within the *PsTCP* family, gene structures and conserved motifs were highly conserved among subfamilies. Comparative homology analysis revealed closer relationships between pea *TCP* genes and dicots (*Arabidopsis*) than monocots (rice). *Cis*-regulatory element analysis suggested roles in growth, hormone response, and stress adaptation. Under salt stress, *PsTCP* genes exhibited divergent expression patterns, with *PsTCP17* showing significant upregulation under extreme stress. Weighted gene co-expression network (WGCNA) and gene ontology (GO) enrichment analyses identified *PsTCP20* as a hub gene regulating photosynthesis and metabolic processes. Tissue-specific expression across 11 pea tissues further highlighted their functional diversity. This study provides insights into the molecular mechanisms of salt stress responses in pea and offers genetic resources for breeding salt-tolerant varieties.

## 1 Introduction

Soil salinity is a significant constraint on plant growth, adversely affecting physiological and biochemical processes. It impairs water and nutrient uptake, leading to reduced crop yields (Ali et al., 2023; Zhang et al., 2023). It induces ion imbalances, osmotic stress, and reactive oxygen species (ROS) accumulation, impairing germination, seedling development, photosynthesis, and water use efficiency (Hamouda et al., 2023; Rashid et al., 2023). Salinity affects 20% of global agricultural land, with projections reaching 50% by 2050, further exacerbated by rising sea levels and reduced precipitation (Otlewska et al., 2020; Ilyas et al., 2024). To mitigate these adverse effects, it is crucial to understand the molecular mechanisms that enable plants to cope with salinity stress. Transcription factors (TFs), as key regulators of gene expression, play a central role in coordinating plant responses to abiotic stresses, including salinity, by modulating the expression of stress-related genes and signaling pathways (Li et al., 2019).

Among the diverse families of transcription factors, the *TCP* family has become a critical player in regulating plant growth and stress adaptation. *TCP* transcription factors are plant-specific proteins that originated early in plant evolution and are named after their founding members: TB1 (TEOSINTE BRANCHED 1) in maize, CYC (CYCLOIDEA) in snapdragon, and PCF (PROLIFERATING CELL FACTOR) in rice (Cubas et al., 1999). Based on structural differences, *TCP* proteins are classified into Class I and Class II. Class I TCPs promote growth by regulating cell division, organ development, and seed germination, while Class II TCPs, characterized by an arginine-rich domain, regulate hormones, defense responses, and branching, often acting as growth inhibitors (Kosugi and Ohashi, 2002; Savadel et al., 2021; Zhang et al., 2021; Yu et al., 2022; Wu et al., 2023). *TCP* transcription factors play pivotal roles in plant responses to abiotic stresses, particularly salinity. Studies have shown that *TCP* genes are involved in stress signal transduction, enabling plants to adapt to saline environments. For example, in rice, OsTCP19 serves as a key regulator that integrates stress responses with developmental pathways, thereby enhancing salt tolerance (Mukhopadhyay and Tyagi, 2015). Similarly, overexpression of *PheTCP9* or *PeTCP10* from *Phyllostachys edul*is in *Arabidopsis thaliana* significantly enhances the salt tolerance in transgenic plants, highlighting their potential positive regulatory functions (Xu et al., 2022). These studies underscore the significant involvement of *TCP* transcription factors in stress response mechanisms, particularly under saline conditions, and point to their potential application in enhancing crop tolerance to salinity and other abiotic stresses.

Pea (*Pisum sativum*) is a self-pollinating, cool-season legume and a globally important crop, valued for its high nutritional content, digestibility, and cost-effective cultivation in temperate regions (Amarakoon et al., 2012; Guindon et al., 2021). Pea seeds are rich in protein, fiber, antioxidants, and essential minerals, with low cholesterol, making them beneficial for reducing cancer and cardiovascular risks, regulating blood glucose, and supporting diabetes management (Mudryj et al., 2014; Poblaciones and Rengel, 2018). Additionally, peas improve soil fertility through nitrogen fixation and are versatile as vegetables, dry peas, or green manure crops (Grela et al., 2017; Singh et al., 2022). Despite its agronomic and nutritional importance, pea production faces significant challenges from abiotic stresses, particularly salinity, which can severely impact yield and quality (Hamouda et al., 2023). To counteract such stresses, plants activate molecular mechanisms, in which transcription factors play a central role.

Although the *TCP* gene family has been extensively studied in various plants (Ling et al., 2020; Li et al., 2022; Pan et al., 2024), its systematic characterization in pea remains unexplored. In this study, we conducted a comprehensive genomic analysis of the *PsTCP* gene family, focusing on their physicochemical properties, gene structures, chromosomal distribution, conserved motifs, *cis*-regulatory elements, and phylogenetic relationships. We also examined their expression patterns under salt stress, revealing dynamic responses across different salt concentrations and time points. Tissue-specific profiling in 11 pea tissues further highlighted their roles in growth, development, and stress adaptation. To explore their functional mechanisms, we constructed protein-protein interaction networks and performed weighted gene co-expression network analysis (WGCNA), which identified key co-expression modules associated with salt tolerance. Additionally, structural predictions of *PsTCP* proteins using AlphaFold3 provided insights into their functional relationships. These findings underscore the critical roles of *PsTCP* genes in pea development and salt stress responses, laying the groundwork for breeding salt-tolerant pea varieties and advancing sustainable agriculture.

## 2 Results

### 2.1 Identification of *TCP* genes in pea and analysis of protein characteristics

By using the Pfam database (ID: PF03634) and performing BLAST alignment with *A. thaliana*, 21 *TCP* gene members were identified in pea. Based on their chromosomal locations, these genes were named *PsTCP1* to *PsTCP21*. In addition, the physicochemical properties of these proteins were analyzed (**Supplementary Table 1**).

The molecular weights of PsTCP proteins range from 54.5 kDa (PsTCP4) to 21.87 kDa (*PsTCP17*). This significant variation in molecular weight suggests that the PsTCP protein family may be involved in diverse biological functions, reflecting structural and functional heterogeneity. The isoelectric points (pI) of the proteins range from 5.88 (*PsTCP19*) to 9.51 (*PsTCP20*). Among them, 9 proteins exhibit weak acidity (pI < 7), while the remaining 12 are alkaline (pI > 7), indicating that the *TCP* family may participate in regulatory processes adapted to various pH environments.

All members of this family are predicted to be unstable (instability index > 40), ranging from 44.14 (*PsTCP2*) to 71.17 (*PsTCP17*), suggesting that these proteins may rely on interactions with other molecules or complexes to maintain functional stability *in vivo*. The aliphatic index (AI) of PsTCP proteins ranges from 51.73 (*PsTCP21*) to 85.26 (*PsTCP2*), with an average of 61.01. Twelve family members exhibit low thermal stability (AI < 60), while the remaining 9 display moderate thermal stability (60 ≤ AI ≤ 90), indicating that the family proteins may have diverse adaptability to varying environmental temperature conditions.

Additionally, all PsTCP proteins exhibit high hydrophilicity (GRAVY < 0), indicating strong solubility in aqueous solutions and a propensity to interact with water molecules. This feature may enhance their functional accessibility within cellular environments. Subcellular localization analysis indicates that *PsTCP9* is localized to the peroxisome, while the remaining 20 are localized to the nucleus. This distribution suggests that TCP proteins may perform distinct functions in specific subcellular environments, particularly emphasizing their roles in transcriptional regulation within the nucleus.

Chromosomal localization analysis revealed that the 21 *PsTCP* genes are unevenly distributed across the 7 chromosomes of pea (**Supplementary Figure 1**, **Supplementary Table 1**). Chromosome 4 the largest number of family members, with 5 genes (*PsTCP9* to *PsTCP13*), while chromosomes 2 (*PsTCP5*) and 7 (*PsTCP21*) each contain only one member. Chromosomes 1 (*PsTCP1* to *PsTCP4*) and 6 (*PsTCP17* to *PsTCP20*) each include 4 members, whereas chromosomes 3 (*PsTCP6* to *PsTCP8*) and 5 (*PsTCP14* to *PsTCP16*) each carry 3 members.

### 2.2 Phylogenetic analysis and conserved motif characterization of the *PsTCP* gene family

**Figure 1:**
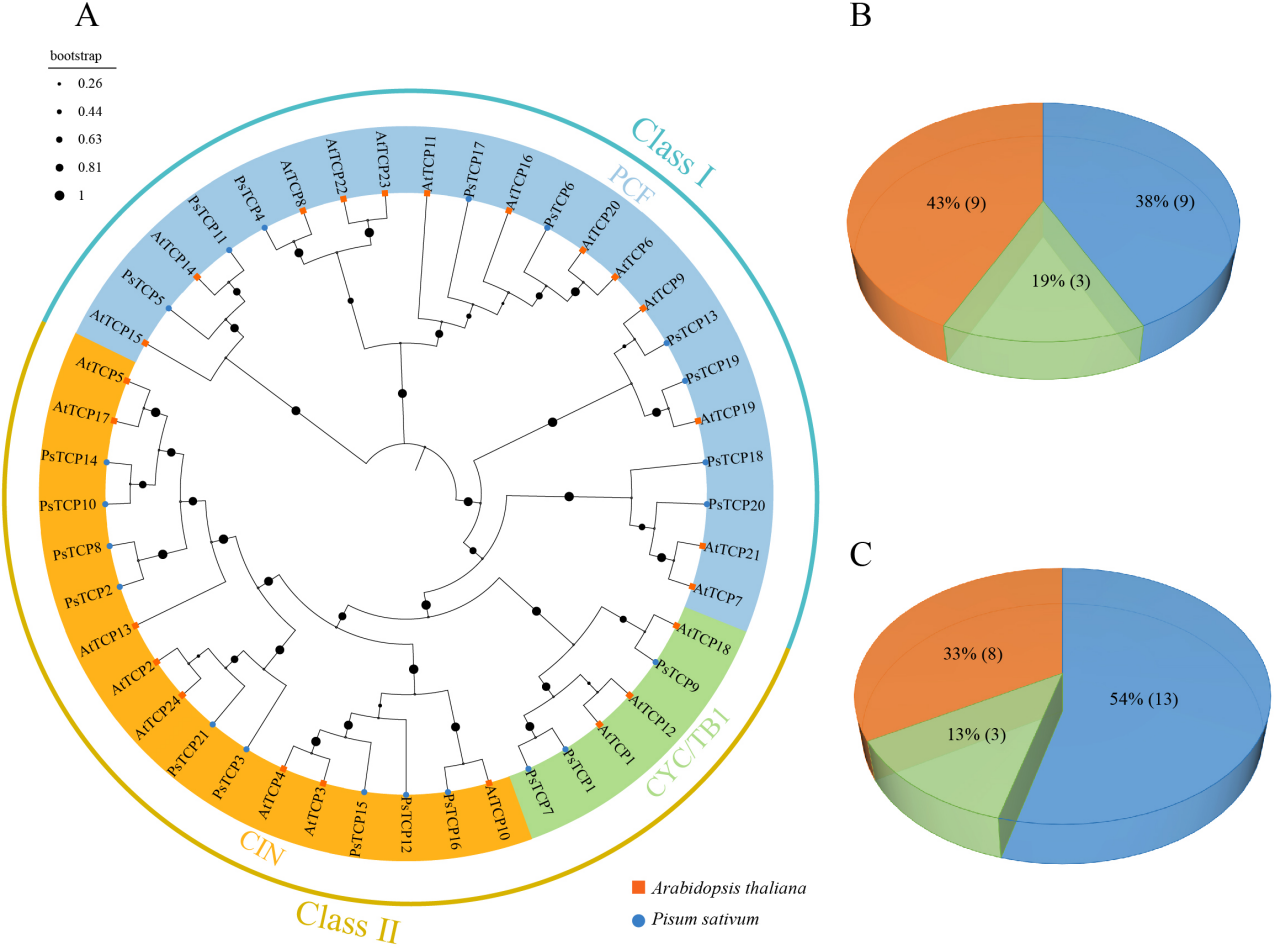
Phylogenetic analysis of TCP proteins. **(A)** The phylogenetic tree of PsTCP and AtTCP proteins, where blue circles represent pea *TCP* genes, and orange squares represent *A*. *thaliana TCP* genes. **(B)** The distribution of the three subfamilies in the *PsTCP* gene family. **(C)** The distribution of the three subfamilies in the *AtTCP* gene family. The colors in the figure represent the following: blue for the PCF subfamily, green for the CYC/TB1 subfamily, and orange for the CIN subfamily.

To investigate the evolutionary relationship of the *PsTCP* family, a phylogenetic tree was constructed using the protein sequences of pea and *A. thaliana* (**Figure 1A**). Based on a multiple sequence alignment of 21 PsTCP and 24 AtTCP proteins, the TCP proteins from both species were classified into two classes and three subfamilies. Class I (PCF subfamily) includes 9 PsTCP (38%) and 13 AtTCP (54%) proteins. Class II is divided into two subgroups: the CYC/TB1 subfamily, which contains 3 PsTCP (19%) and 3 AtTCP (13%) proteins, and the CIN subfamily, which includes 9 PsTCP (43%) and 8 AtTCP (33%) proteins (**Figures 1B, C**).

**Figure 2:**
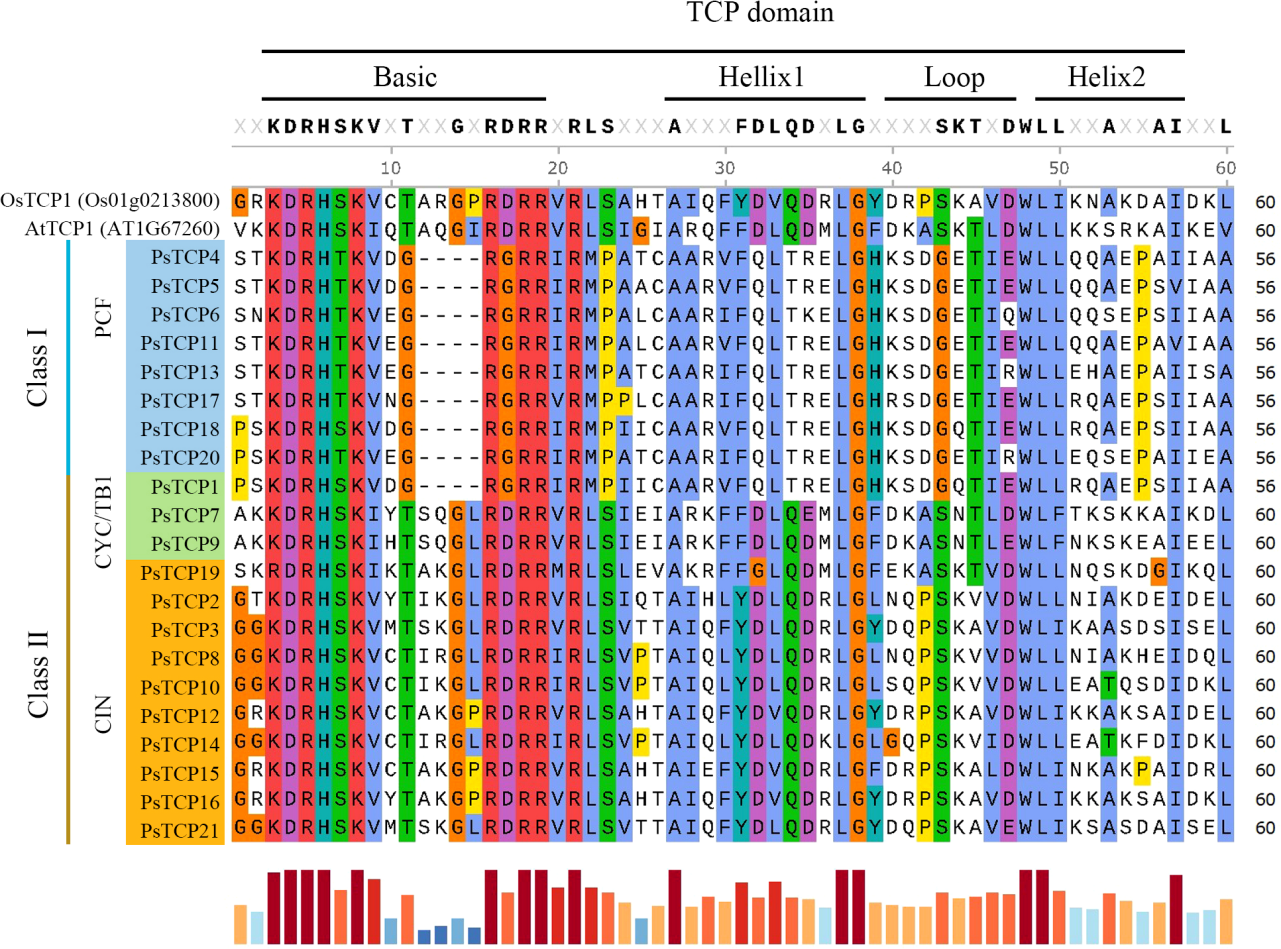
Domain multiple sequence alignment of PsTCP proteins. *OsTCP1* represents the TCP protein from rice, and *AtTCP1* represents the TCP protein from *A. thaliana*. The bar chart at the bottom indicates the level of sequence conservation, with longer red bars representing higher conservation and shorter blue bars indicating lower conservation.

Analysis of the 21 PsTCP proteins revealed four conserved motifs across TCP proteins from different species: the base region, helix I, loop, and helix II, with high conservation was observed within the same subfamily (**Figure 2**). Class I TCP proteins lack four amino acids in the base region compared to Class II TCP proteins, leading to distinct DNA-binding specificities (Class I: GGNCCAC; Class II: GTGGNCCC). Despite these differences, structural conservation remains high, enabling clear differentiation between subfamilies. The base region exhibits the highest conservation, with key amino acids like lysine (K), aspartic acid (D), arginine (R), and histidine (H) being completely conserved. These residues are likely crucial for protein function, while the helix region shows moderate conservation and the loop region the least, suggesting a functional hierarchy among the motifs.

### 2.3 Structural analysis of the *PsTCP* transcription factor family proteins

The three-dimensional structures of all *PsTCP* family proteins were predicted using AlphaFold3, and the results were visualized with color-coded confidence levels to indicate the reliability of the structural models (**Supplementary Figure 2**). The α-helix regions consistently demonstrated the highest prediction confidence across all analyzed proteins, reflecting their structural stability and evolutionary conservation within functional domains.

**Figure 3:**
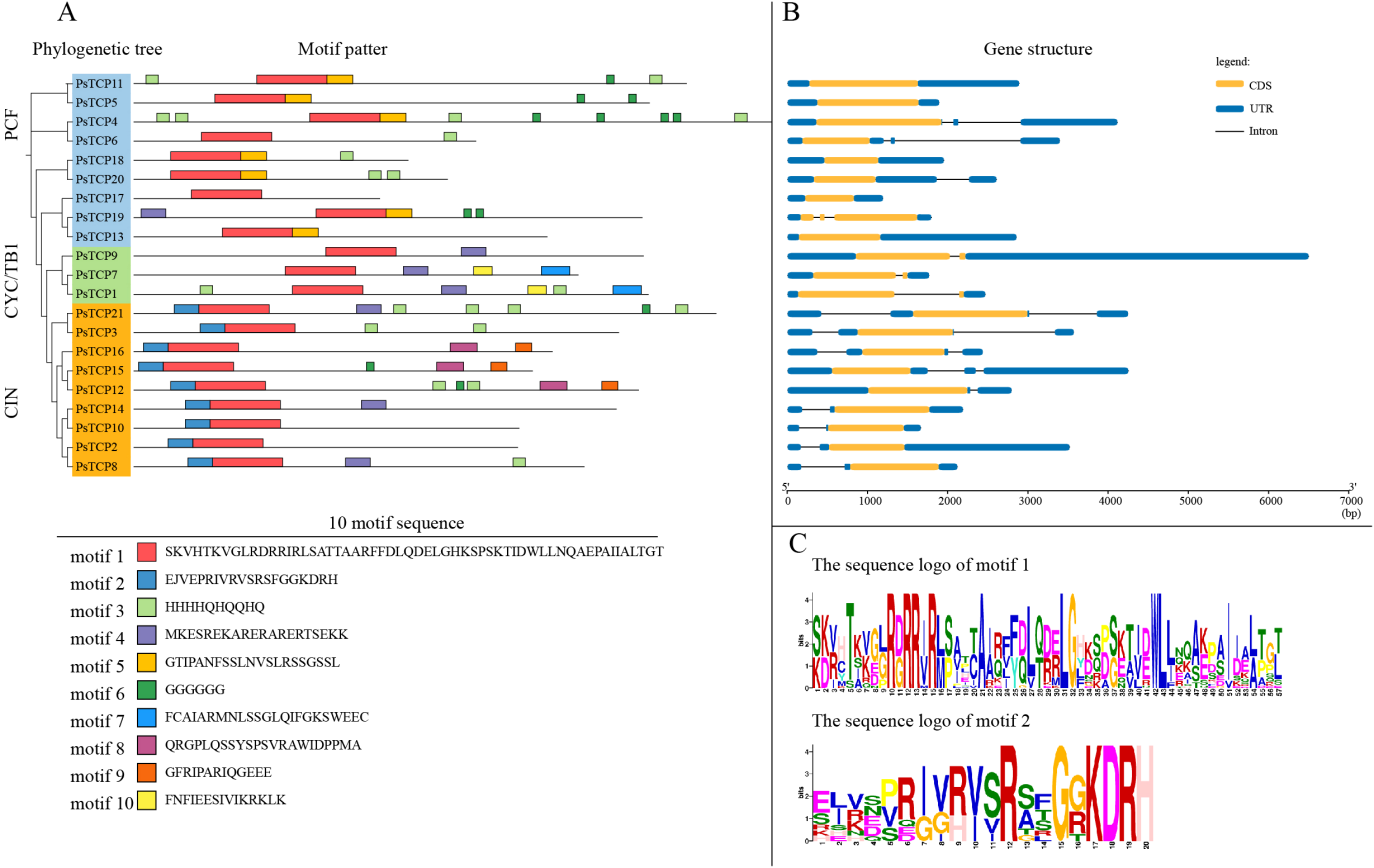
Conserved domains, motif analysis, and gene structure analysis of *PsTCP* genes. **(A)** The phylogenetic tree of *PsTCP* and the distribution of 10 motifs, with motifs 1 to 10 represented by rectangles in different colors. **(B)** The gene structure of *PsTCP*, where orange boxes represent coding sequences (CDS) black lines represent introns, and blue boxes represent the 5’ and 3’ untranslated regions. **(C)** The sequence identification of motifs 1 and 2, with colored letters representing the specific amino acid sequences of the motifs.

We further analyzed the conserved motif sequences and identified 10 conserved motifs, named motifs 1 to 10 (**Figure 3A**). The results indicate that these motifs exhibit a certain degree of conservation across different subfamilies. All *PsTCP* family members contain motif 1, suggesting that this motif is highly conserved in *PsTCP* proteins and may be closely related to the fundamental functions of the family. Motif 2 is present in all CIN subfamily members and is located at the N-terminal of the protein, which could be a distinguishing feature of the CIN subfamily compared to other subfamilies. This motif may be related to its role in specific biological processes, such as flower development and embryo development (Rath et al., 2022). Motif 5 is found only in a subset of members of the PCF subfamily, while motif 7 is present only in certain members of the CYC/TB1 subfamily. The presence of these specific motifs suggests structural differences among members of the different *PsTCP* subfamilies, reflecting potential functional diversity. These structural specificities provide important clues for further research into the function, evolution, and potential roles of *PsTCP* family members in plant growth, development, and stress responses.

Additionally, we analyzed the gene structure of the *PsTCP* family (**Figure 3C**). The results revealed that *PsTCP11*, *PsTCP5*, *PsTCP18*, *PsTCP17* and *PsTCP13* lack intronic regions, which may be related to certain mechanisms in genome evolution, such as the activity of transposons (Venner et al., 2009). Transposons are mobile genetic elements that can insert themselves at different locations in the genome.

### 2.4 Synteny analysis and selective pressure evaluation of the *PsTCP* gene family

**Figure 4:**
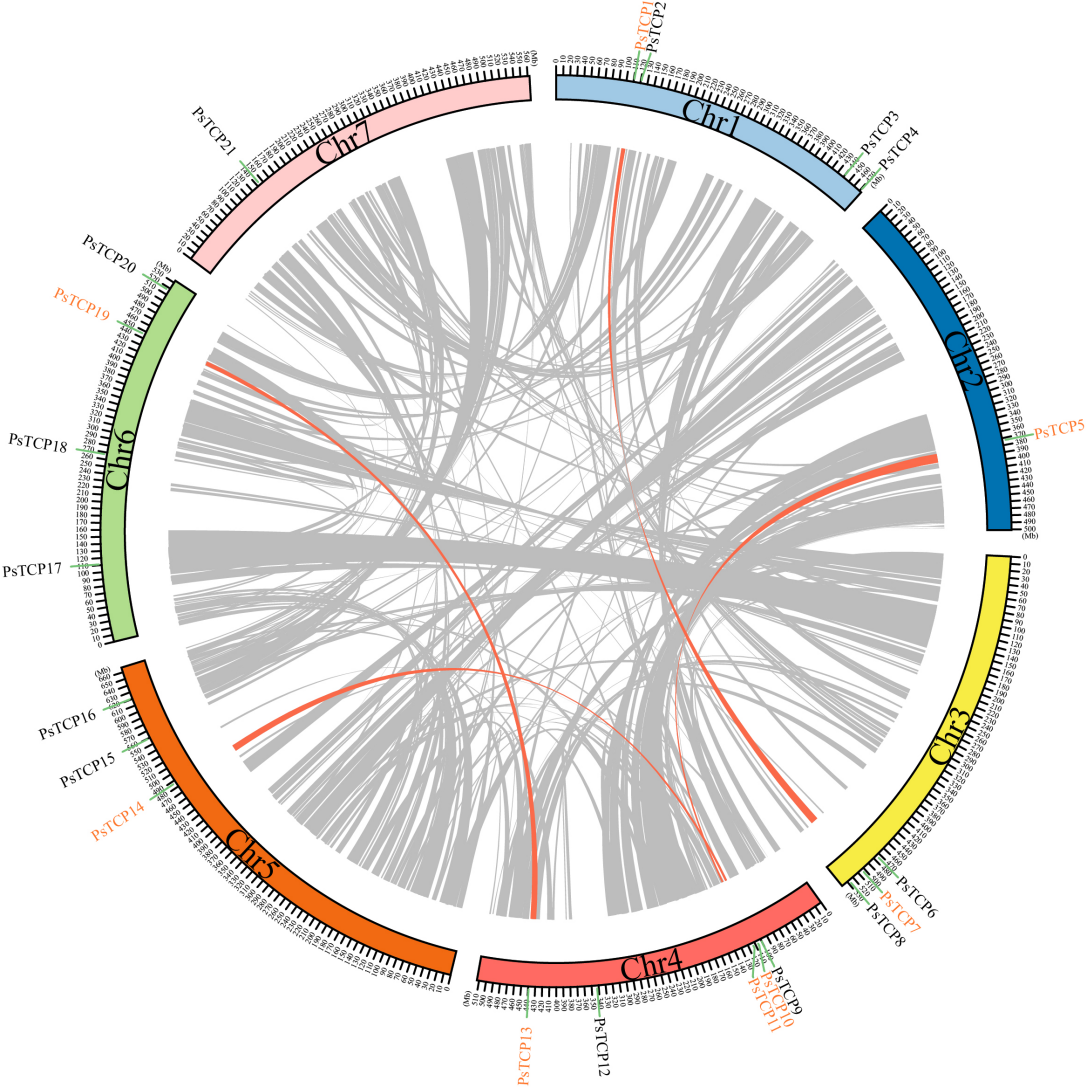
Genomic distribution, duplication events, and synteny analysis of PsTCP genes. The figure displays the synteny analysis of the *PsTCP* gene family in pea, where gray lines represent syntenic regions in the pea genome, and orange lines indicate duplicated *PsTCP* gene pairs. Genes marked in orange represent *PsTCP* genes with duplication events. Chromosome numbers are labeled within the boxes for each chromosome.

Synteny analysis revealed that the 21 *PsTCP* genes are unevenly distributed across 7 chromosomes in the pea genome (**Figure 4**; **Supplementary Figure 1**). Among the 2,711 syntenic events identified (**Supplementary Table 2**; **Figure 4**), four *PsTCP* gene pairs-*PsTCP10*-*PsTCP14*, *PsTCP13*-*PsTCP19*, *PsTCP1*-*PsTCP7*, and *PsTCP5*-*PsTCP11*-underwent segmental duplication (**Table 1**; **Figure 4**). Notably, no duplication events were observed for other *PsTCP* family members, indicating that segmental duplications are limited to a small subset of genes.

**Table 1:** *Ka*, *Ks*, and *Ka/Ks* of duplicated gene pairs of *PsTCP* family genes.

Duplicated Gene Pairs	*Ka*	*Ks*	*Ka/Ks*	Duplicated Type
*PsTCP10-PsTCP14*	0.411272	2.57386	0.159788	segmental
*PsTCP13-PsTCP19*	0.553198	2.21351	0.249919	segmental
*PsTCP1-PsTCP7*	0.283056	1.02532	0.276065	segmental
*PsTCP5-PsTCP11*	0.427514	2.88987	0.147935	segmental

The *Ka/Ks* (Non-Synonymous/Synonymous) ratio is a critical indicator for assessing selective pressure on protein-coding genes. Our analysis revealed that the *Ka/Ks* ratios of all duplicated *PsTCP* gene pairs are below 0.3 (**Table 1**), indicating that these gene pairs have undergone purifying selection during evolution. Among the analyzed pairs, *PsTCP1-PsTCP7* exhibited the highest *Ka/Ks* ratio (0.276065), while *PsTCP5-PsTCP11* showed the lowest ratio (0.147935). This variation in *Ka/Ks* ratios suggests differences in the intensity of purifying selection across gene pairs, potentially reflecting subtle variations in evolutionary constraints or functional divergence within the *PsTCP* gene family.

To explore the evolutionary relationships of the *PsTCP* gene family across different species, we conducted a synteny analysis between pea, the monocot rice, and the dicot *A. thaliana* (**Supplementary Figure 3A**). The results showed that pea and *A. thaliana* (dicot) shared a stronger gene synteny, with 338 pairs of syntenic genes, including 10 pairs of *PsTCP* genes. In contrast, the synteny between pea and rice (monocot) was weaker, with only 193 pairs of syntenic genes, and of which only four pairs were related to the *PsTCP* gene family. The synteny between *A. thaliana* and rice was the weakest, with only 150 pairs of syntenic genes, and 3 pairs related to the *PsTCP* gene family. This pattern reflects the genetic conservation between dicots, suggesting that dicots may have retained more shared genetic features during evolution, resulting in higher similarity in gene structure and function between these species. On the other hand, the significant differences in genome structure and evolutionary paths between monocots (rice) and dicots (such as pea and *A. thaliana*) reflect the early divergence of these species, leading to weaker synteny of the *PsTCP* gene family between them. These synteny results not only provide important insights into the evolutionary history of the *PsTCP* gene family across species but also help reveal the dynamics of gene amplification and independent evolution.

The *Ka/Ks* analysis further supports these findings (**Supplementary Figure 3B**). The lower *Ka/Ks* ratios between pea and *A. thaliana* compared to those between pea and rice indicate stronger purifying selection among dicots, which may contribute to the preservation of essential genetic functions. The higher *Ka/Ks* values observed between monocots and dicots underscore their more distinct evolutionary trajectories, shaped by genome divergence and lineage-specific adaptations.

### 2.5 Analysis of *cis*-regulatory elements in the promoter regions of *PsTCP* genes

**Figure 5:**
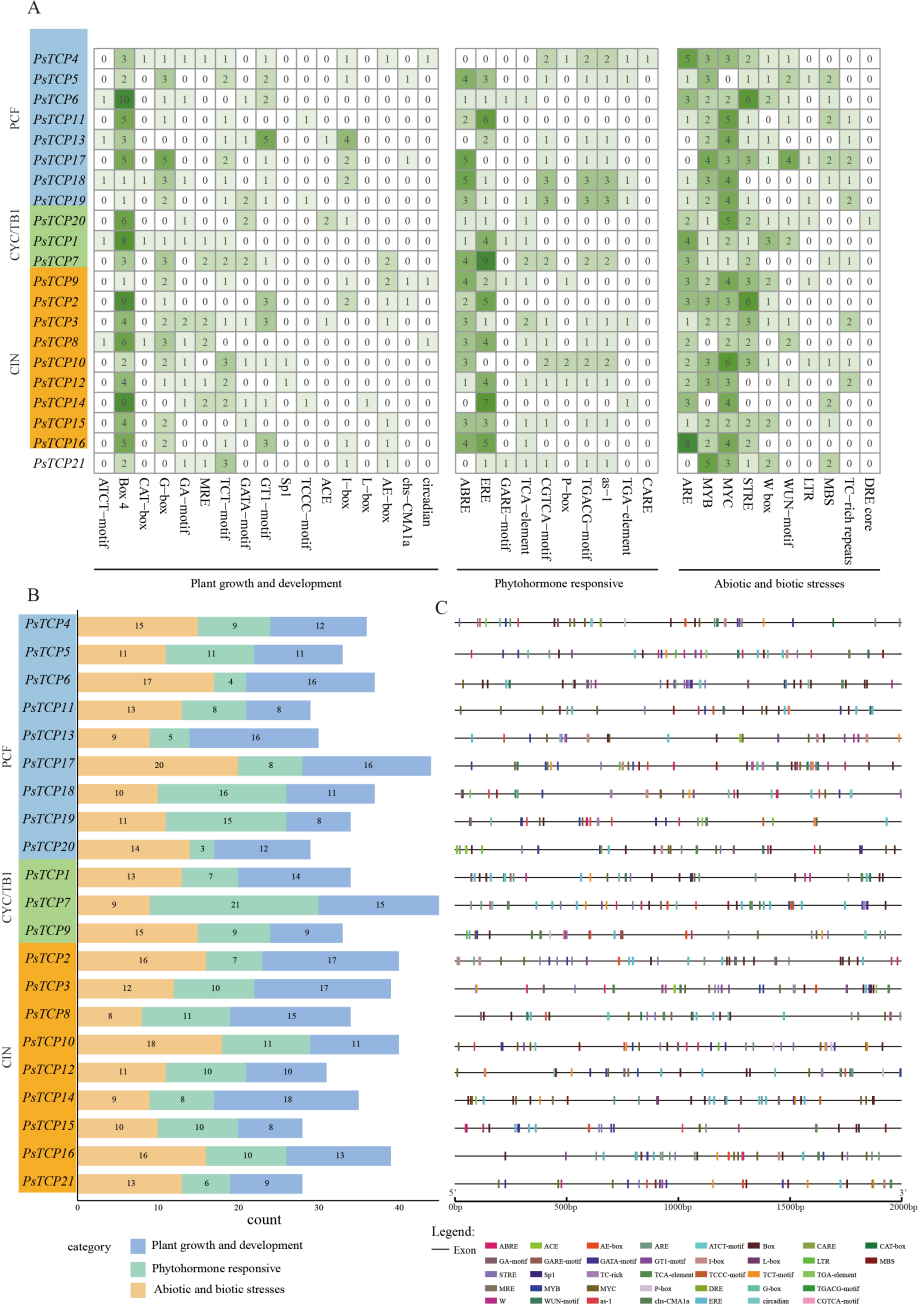
*Cis*-regulatory element analysis of the *PsTCP* gene promoter regions. The 2000 bp upstream sequences of each *PsTCP* family gene were extracted as promoter regions, and *cis*-regulatory elements were predicted using the PlantCARE database. Based on their functions related to plant growth and development, phytohormone responsive, and abiotic and biotic stress responses, the *cis*-regulatory elements were classified into three major categories. **(A)** A heatmap showing the number of functional *cis*-regulatory elements in each *PsTCP* gene across the three subfamilies. The darker the color, the higher the number of elements; **(B)** Statistical analysis of the number of *cis*-regulatory elements in each *PsTCP* gene, categorized by function: plant growth and development, phytohormone responsive, and abiotic and biotic stress response; **(C)** Specific distribution of *cis*-regulatory elements in the promoter regions.

To gain deeper insight into the transcriptional regulatory mechanisms of the *PsTCP* gene family, we analyzed the *cis*-regulatory elements in the upstream 2000 bp sequence of its promoter regions. Using the PlantCARE database, we analyzed the promoter sequences of 21 *PsTCP* genes and identified 37 *cis*-regulatory elements, which were classified into three main functional categories: plant growth and development (45.9%, 17 elements), phytohormone responsive (27%, 10 elements), and abiotic and biotic stress responses (27%, 10 elements) (**Figure 5A**). This distribution reveals that the *PsTCP* gene family may respond to a variety of physiological needs during different biological processes through different *cis*-regulatory elements.

The number of functional *cis*-regulatory elements varies among different genes. For example, *PsTCP7* contains the highest number of functional *cis*-regulatory elements (45 elements), while *PsTCP15* contains the fewest (28 elements). This suggests that differences in the number of *cis*-regulatory elements may reflect the different functional needs of the genes under various physiological conditions. For instance, *PsTCP2* and *PsTCP3* contain the highest number of plant growth and development-related *cis-*regulatory elements (17 elements each), while *PsTCP15*, *PsTCP11*, and *PsTCP19* contains the fewest (8 elements). Similarly, *PsTCP7* contains the most phytohormone responsive *cis-*regulatory elements (21 elements), while *PsTCP20* contains the fewest (only 3 elements). For abiotic and biotic stress responses, *PsTCP17* contains the most *cis*-regulatory elements (20 elements), while *PsTCP8* contains the fewest (8 elements) (**Figure 5B**).

### 2.6 Protein-protein interaction network of the *PsTCP* gene family

To further investigate the potential functions of the *PsTCP* gene family in pea, we predicted their protein-protein interaction (PPI) networks (**Supplementary Figure 4**). Among the 21 *PsTCP* family members, 16 genes were grouped into two distinct interaction modules, while *PsTCP2*, *PsTCP3*, *PsTCP10*, *PsTCP11*, and *PsTCP12* did not form interactions with other *PsTCP* genes. These non-interacting genes may engage in regulatory relationships with genes outside the *PsTCP* family.

The first module consisted of 13 *PsTCP* genes, with *PsTCP5*, *PsTCP14*, and *PsTCP13* exhibiting the highest connectivity, suggesting their central roles in the network. The second module included only three genes, connected through *PsTCP15* as the core node. These two modules highlight the existence of subfamily-specific functional units within the *PsTCP* gene family, which may reflect distinct roles in pea growth and stress response pathways. High-connectivity genes, such as *PsTCP5*, *PsTCP14*, and *PsTCP13*, likely act as regulatory hubs that mediate intra-family interactions and may also participate in broader signaling pathways. Previous studies have shown that hub genes often play critical roles in maintaining network stability and facilitating adaptive responses to environmental stresses (Deng et al., 2018). These findings provide new insights into the structural and functional organization of the *PsTCP* gene family, suggesting diverse roles in pea development and stress adaptation.

### 2.7 The expression pattern of the *PsTCP* gene family in different tissues of pea

To investigate the tissue-specific expression patterns of *PsTCP* genes, RNA-seq analysis was performed on 11 tissues from two pea varieties: Zhongwan 6 and Yunwan127. Zhongwan 6 tissues included white flower (WF), normal stipule (NS), root (R), green pod (GP), stem (S), fresh seed (FS), tendril (T), and imparipinnate leaf (IL), while Yunwan127 tissues included light purple vexilla (LPV), dark purple wing (DPW), and purple pod (PP). The Yunwan127 variety was selected due to its distinctive purple pigmentation in flowers and pods. After stringent quality control, all samples met the established criteria, yielding a total of 149.11 Gb of high-quality sequencing data (**Supplementary Table 3**). The RNA-seq reads were aligned to the reference genome, and expression profiles of *PsTCP* genes were obtained (**Supplementary Table 6**). The expression patterns of the *PsTCP* family across tissues were visualized (**Supplementary Figure 6**).

In the analysis, *PsTCP15* was not detected in any tissue, which may indicate its activation under specific stress conditions, tissue-specific regulation, or potential issues such as gene annotation errors or insufficient sequencing depth. In contrast, *PsTCP1* exhibited expression in both WF and LPV tissues, while *PsTCP9* showed broad expression across multiple tissues, including R, S, T, LPV, WF, and DPW (**Supplementary Figure 6**).

These findings highlight the tissue-specific expression heterogeneity of the *PsTCP* gene family, suggesting their diverse roles in plant growth, development, and stress responses. For example, certain *PsTCP* genes may regulate developmental processes in specific tissues, while others may modulate responses to environmental stimuli or hormonal signals. The tissue-specific expression patterns provide valuable insights into the functional diversification of *PsTCP* genes and offer potential targets for improving stress tolerance, such as salt resistance, in pea varieties.

### 2.8 Expression pattern of the *PsTCP* gene family under salt stress

**Figure 6:**
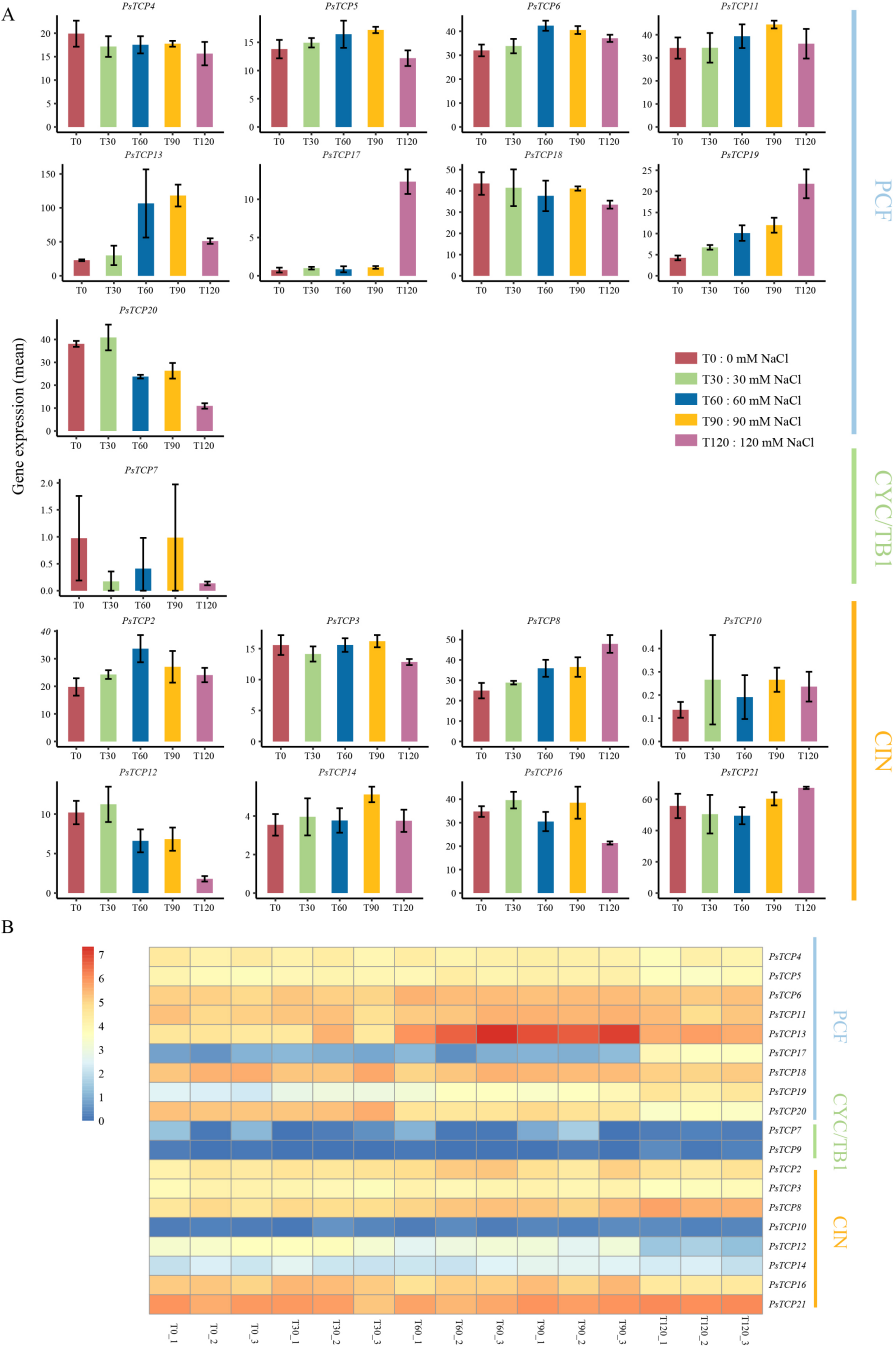
Expression profiles of *PsTCP* genes under different NaCl concentrations. **(A)** Bar chart showing the expression levels. Gene names are labeled above each subplot. The x-axis represents the expression levels, and the y-axis represents different treatment groups. The bar chart displays the average expression levels of the samples in each treatment group, with error bars representing the standard deviation (SD). **(B)** Heatmap of expression levels. Red indicates high expression, while blue indicates low expression. Gene names are shown on the right side of the heatmap, and treatment samples are displayed below. The classification of the three subfamilies is labeled on the right. Genes with expression levels below 1 in all treatments have been filtered out.

To investigate the function of the *PsTCP* gene family under salt stress, we designed two experimental sets. The first set involved treating plants with different concentrations of NaCl (T0: 0 mM NaCl, T30: 30 mM NaCl, T60: 60 mM NaCl, T90: 90 mM NaCl, T120: 120 mM NaCl) for 3 days. The second set used 100 mM NaCl treatment at different time points (0 hours: H0, 1 hour: H1, 3 hours: H3, 6 hours: H6, 12 hours: H12, 24 hours: H24, 48 hours: H48). Each experiment included three biological replicates.After quality control of the sequencing data, all samples met the established criteria, yielding a total of 159.42G of high-quality data (**Supplementary Table 3**). These data were aligned to the reference genome to obtain expression data for the *PsTCP* genes (**Figure 6**; **Supplementary Figure 5**; **Supplementary Tables 4**, **5**). Among the 21 *PsTCP* genes, 18 showed expression in pea leaves, with a TMM value greater than 1 (**Figure 6B**; **Supplementary Figure 5B**), with *PsTCP13* and *PsTCP21* exhibiting higher expression levels, while *PsTCP7* and *PsTCP10* showed lower expression levels (**Figure 6**). In the experiments with different NaCl concentrations, the expression levels of *PsTCP19* and *PsTCP8* increased with rising NaCl concentrations, suggesting that these two genes have a positive response to salt stress and may play a promotive role in the early stages of salt stress. In contrast, the expression of *PsTCP4*, *PsTCP18*, *PsTCP20*, and *PsTCP12* decreased with increasing NaCl concentrations, indicating that these genes might be suppressed under high salt stress, potentially due to their negative regulatory role in salt stress (**Figure 6**).

Further analysis demonstrated that under salt stress conditions, multiple *TCP* genes in pea exhibited diverse expression trends. Specifically, the expression levels of *PsTCP5, PsTCP6, PsTCP11, PsTCP13, PsTCP2* and *PsTCP14* initially showed an upward trend as the NaCl concentration gradually increased. After reaching a certain peak value, they turned to a downward trend, presenting an overall dynamic change pattern of first rising and then falling. In contrast, the expression level change trend of *PsTCP21* was exactly the opposite. As the NaCl concentration increased, the expression level of this gene first decreased. When the concentration continued to increase to a certain stage, its expression level then increased instead, showing a unique change pattern of first falling and then rising (**Figure 6**). Notably, *PsTCP17* showed a significant increase in expression under high salt concentration (120 mM NaCl), suggesting that it may play a special regulatory role under extreme salt stress conditions, possibly involved in a stronger stress response mechanism that helps the plant adapt to severe salt stress environments (**Figure 6**). After the 100 mM NaCl treatment, the expression levels of *PsTCP5*, *PsTCP11*, *PsTCP13*, *PsTCP3*, *PsTCP10*, and *PsTCP16* showed a pattern of initial increase followed by a decrease over the time course (0–48 hours). In contrast, the expression levels of *PsTCP18* and *PsTCP7* exhibited a decrease followed by an increase (**Supplementary Figure 5**). Notably, *PsTCP17* showed a significant increase in expression after prolonged salt stress, and its expression pattern was similar to the trend observed under different NaCl concentrations (**Supplementary Figure 5**). These findings highlight the significant role of the *PsTCP* gene family in the adaptive responses of pea to salt stress. The distinct expression patterns observed among *PsTCP* genes suggest their involvement in key regulatory mechanisms that enable the plant to cope with varying salt conditions. This study provides important insights into the molecular functions of *PsTCP* genes under salt stress and identifies potential genetic targets for breeding salt-tolerant pea varieties.

### 2.9 The regulatory network and functional analysis of the *PsTCP* gene family in response to salt stress in pea

**Figure 7:**
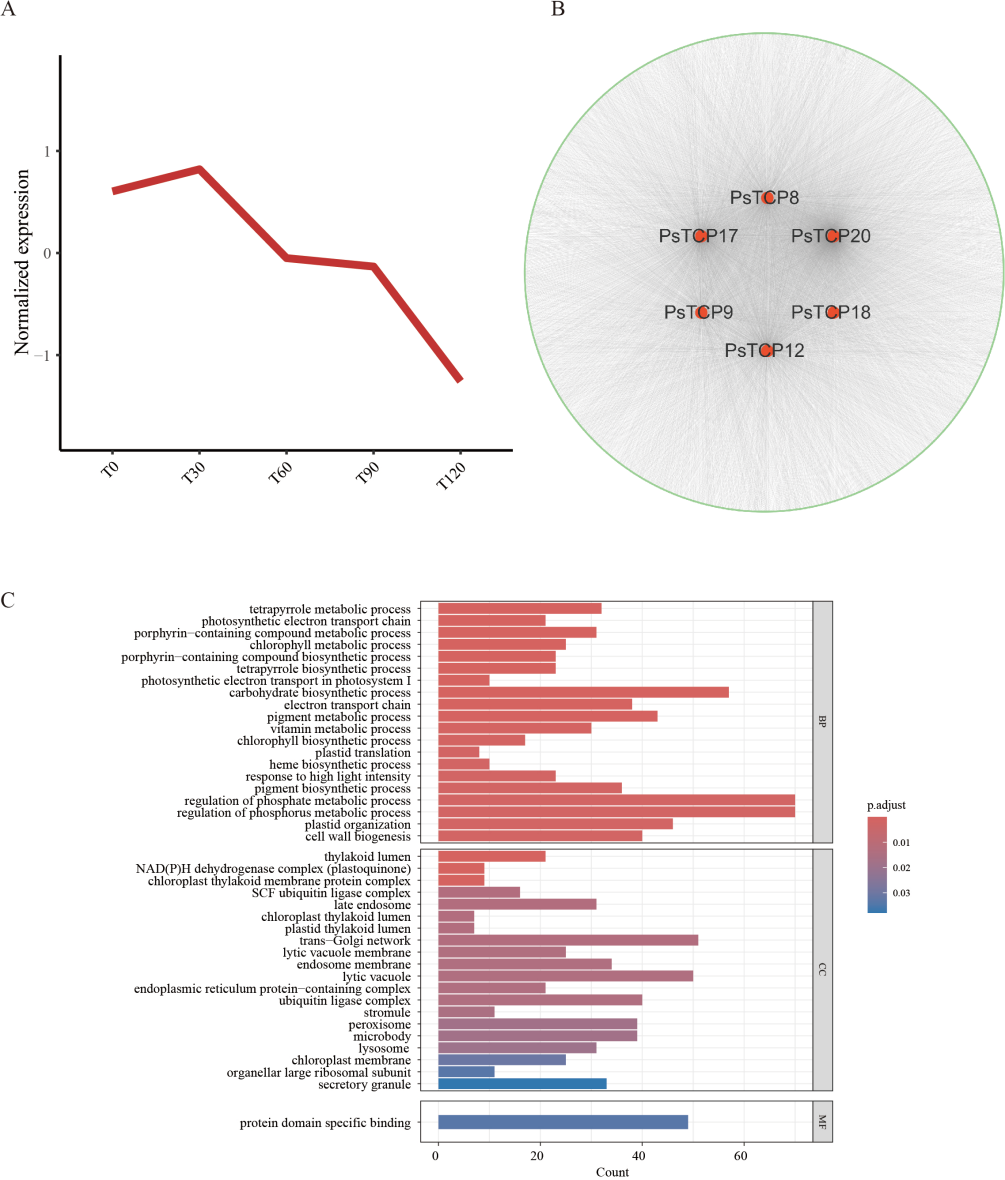
WGCNA and GO enrichment analysis **(A)** Expression patterns of genes in the blue module. **(B)** Co-expression network of *PsTCP* family genes in the blue module, where red nodes represent *PsTCP* family genes, green nodes represent other module genes co-expressed with *PsTCP* genes, and gray lines indicate co-expression relationships between genes. **(C)** GO functional enrichment analysis of genes co-expressed with *PsTCP* family genes in the blue module.

To further investigate the critical role of *PsTCP* genes in pea salt tolerance, we constructed a WGCNA network based on transcriptome data from pea plants treated with different concentrations of NaCl. *PsTCP* genes were distributed across nine co-expression modules (**Supplementary Table S7**), with the blue module containing the highest number of *PsTCP* genes (six genes) (**Figure 7B**; **Supplementary Table 7**). This module showed a strong correlation (r = 0.92, p = 2e−06) with different salt treatments, making it the focus of our analysis. The blue module consisted of 5,960 co-expressed genes (**Supplementary Table S8**). By comprehensively analyzing the expression patterns of these genes (**Figure 7A**), the co-expression network (**Figure 7B**), and functional enrichment results (**Figure 7C**), we identified the central role of *PsTCP* genes within the network and their potential regulatory functions under salt stress.

GO enrichment analysis revealed that genes co-expressed with *PsTCP* were significantly enriched in biological processes related to photosynthesis and metabolism, including “photosynthetic electron transport in photosystem I”, “chlorophyll metabolic process”, “tetrapyrrole biosynthetic process”, “plastid translation”, and “electron transport chain” (**Figure 7C**). These results suggest that the *PsTCP*-co-expressed network is closely associated with key physiological functions critical for energy metabolism and material conversion in pea.

Expression trend analysis further showed that genes in this network were significantly upregulated under low salt stress (T30), but their expression levels gradually decreased as NaCl concentrations increased (**Figure 7A**). This trend implies that these genes may actively participate in early salt stress responses by enhancing photosynthetic and metabolic activities. However, their downregulation under higher NaCl concentrations likely reflects the inhibitory effects of severe salt stress on photosynthesis and metabolism, suggesting an adaptive shift in metabolic priorities under stress conditions.

In the co-expression network (**Figure 7B**), *PsTCP* family genes exhibited varying degrees of connectivity, with *PsTCP20* showing the highest degree (2742), followed by *PsTCP17* (1415), *PsTCP12* (1027), *PsTCP8* (851), *PsTCP9* (440), and *PsTCP18* (63). This highlights *PsTCP20* as a core hub gene within the regulatory network, potentially playing a pivotal role in coordinating pea responses to salt stress. *PsTCP17* and *PsTCP12*, with relatively high connectivity, might also function synergistically with other genes to regulate physiological and biochemical pathways involved in salt tolerance.

Taken together, these findings indicate that *PsTCP* genes, particularly *PsTCP20*, are integral to salt tolerance in pea by regulating photosynthesis and metabolic pathways through their co-expression network. This study provides insights into the regulatory mechanisms of *PsTCP* genes under salt stress and offers a theoretical basis for further exploring salt tolerance networks in pea.

## 3 Materials and methods

### 3.1 Identification and physicochemical properties of the *PsTCP* gene family in pea

The reference genome sequence of pea was retrieved from NCBI (PRJNA1042956) (Liu et al., 2024), while the genome data of *A. thaliana* and rice were obtained from the Phytozome database (https://phytozome-next.jgi.doe.gov/). To identify the *PsTCP* gene family, the Hidden Markov Model (HMM) profile of the *TCP* domain (Pfam ID: PF03634) was downloaded from the Pfam database (El-Gebali et al., 2018). Potential *PsTCP* genes were identified using HMMER software (https://github.com/EddyRivasLab/hmmer) with default parameters. For further validation, the TCP protein sequences of A. thaliana were aligned with the pea protein sequences using BLASTP, with an E-value cutoff of 1e-5 and other parameters set to default. Candidate sequences with ≥30% identity to known TCP proteins were retained, and cross-referencing with Pfam search results confirmed the identification of 21 *PsTCP* genes, designated *PsTCP1* to *PsTCP21* based on their genomic locations.

To assess the structural conservation of PsTCP proteins, conserved domain analysis was performed using the Batch CD-Search tool with default parameters (Wang et al., 2022a). The physicochemical properties of the PsTCP proteins, including molecular weight, theoretical isoelectric point (pI), instability index, aliphatic index, and grand average of hydropathicity (GRAVY), were predicted using TBtools-II (Chen et al., 2023). Subcellular localization predictions were conducted using WoLF PSORT (https://wolfpsort.hgc.jp/).

### 3.2 Phylogenetic analysis

Using the Muscle5 (v5.1) software with default parameters (Edgar, 2022), we aligned the amino acid sequences of *PsTCP* and *AtTCP* proteins. Based on the alignment results, we constructed a rooted maximum likelihood (ML) phylogenetic tree using FastTree (v2.1.11) (Price et al., 2010), applying the JTT (Jones-Taylor-Thornton) model with default settings for branch length optimization and calculating SH-like local support values. For analysis and visualization, the tree was rendered using the interactive Tree of Life (iTOL) tool (Letunic and Bork, 2021), where the colors and styles of the branches were adjusted to distinguish TCP proteins from different groups.

### 3.3 Protein 3D structure, motif, and gene structure analysis

The three-dimensional (3D) structures of PsTCP proteins were predicted using the SWISS-MODEL server (https://swissmodel.expasy.org/), complemented by structural models retrieved from the AlphaFold database for comprehensive visualization. For motif identification, the Multiple Em for Motif Elicitation (MEME) suite (Bailey et al., 2009) was employed with the “any number of repetitions” (anr) option, configured to identify 10 motifs with a length range of 6–200 amino acids. The input data consisted of the full-length amino acid sequences of PsTCP proteins. Gene structures were visualized using the Gene Structure Display Server (GSDS) 2.0 (Hu et al., 2014), generating diagrams to highlight structural differences among *PsTCP* family members.

### 3.4 Chromosomal localization, gene duplication, synteny relationships, and *Ka/Ks* calculation of homologous genes

The chromosomal locations of *PsTCP* genes were mapped using the pea genome annotation file and visualized with MapChart software (Voorrips, 2002). Synteny analysis was performed at both intra- and inter-species levels. For intra-species synteny, MCScanX (Wang et al., 2012) was employed with default parameters to generate synteny files, and the results were visualized using Circos (Krzywinski et al., 2009). For inter-species synteny analysis, the JCVI toolkit (v1.0.11) (Tang et al., 2008) was utilized to preprocess data, including file format optimization, removal of duplicate entries, and matching of CDS and protein sequences. The jcvi.compara.catalog module facilitated pairwise genome comparisons among *A.thaliana*, pea, and rice to identify syntenic blocks and extract homologous gene pairs. Chromosomal homology relationships were visualized using the jcvi.graphics.karyotype module. Gene and sequence extraction, as well as filtering, were performed using SeqKit (v2.4.0) (Shen et al., 2016).

For the identified homologous gene pairs, *Ka*, *Ks*, and *Ka/Ks* ratio were calculated using ParaAT (v2.0) (Zhang et al., 2012). Protein sequences were aligned with Muscle (Edgar, 2022), and the alignments were combined with corresponding nucleotide sequences for downstream analysis. *Ka* and *Ks* values were computed using KaKs_Calculator 2.00 (Wang et al., 2010) under the default model, with abnormal values (*Ka*/*Ks* > 2) filtered out. Homologous gene pairs were categorized into three species combinations: A. thaliana vs. pea, pea vs. rice, and *A. thaliana* vs. rice.

### 3.5 *Cis*-acting element prediction

The promoter regions of *PsTCP* genes were defined as the 2000 bp upstream sequences, which were extracted using the -up-stream parameter in SeqKit (Shen et al., 2016). These sequences were subsequently analyzed with PlantCARE (Lescot et al., 2002) to identify and annotate *cis*-acting elements, including their positions and counts. Functional filtering and statistical analysis of the *cis*-acting elements were performed using the heatmap and ggplot2 packages in R. Finally, the distribution of *cis*-acting elements was visualized using the Gene Structure Display Server (GSDS) 2.0 (Hu et al., 2014).

Using the -up-stream parameter of SeqKit software (Shen et al., 2016), 2000bp upstream sequences of the *PsTCP* genes were extracted as promoter regions to identify and predict *cis*-acting elements. The sequences were then analyzed using PlantCARE (Lescot et al., 2002) to determine the positions and numbers of *cis*-acting elements. Functional filtering, statistical analysis, and visualization of the *cis*-acting elements were carried out using the heatmap3 (https://github.com/slzhao/heatmap3) and ggplot2 (https://ggplot2.tidyverse.org/) packages. Finally, the distribution of *cis*-acting elements was visualized using the GSDS (v2.0) platform (https://gsds.gao-lab.org/).

### 3.6 Protein-protein interaction network prediction

The STRING database (https://string-db.org/) was used to predict the protein-protein interactions of *PsTCP* proteins. The *PsTCP* protein sequences were input, with the confidence threshold set to 0.4 and other parameters kept at their default settings. The predicted results were then visualized using Cytoscape (v3.10.3) (Shannon et al., 2003), and the node colors and sizes were adjusted based on the degree of interaction.

### 3.7 The study of transcriptional expression patterns of *PsTCP* genes under salt stress and in different pea tissues

To study the response mechanism of *PsTCP* genes in pea under salt stress, two experimental sets were designed. First, pea seeds were transferred to hydroponic boxes filled with deionized water three days after germination, with no additional nutrients. All seedlings were grown under controlled conditions: a constant temperature of 26°C, relative humidity of 50%, a 16-hour light cycle (200 µmol photons m^-2^ s^-2^), and an 8-hour dark period. After one week of growth, salt stress treatments were applied, and fully expanded primary leaves were collected for sampling. Each treatment group included three biological replicates.

In the first experiment, different NaCl concentrations were applied: T0 (0 mM NaCl), T30 (30 mM NaCl), T60 (60 mM NaCl), T90 (90 mM NaCl), and T120 (120 mM NaCl). Samples were collected after 72 hours of treatment. In the second experiment, a fixed NaCl concentration of 100 mM was used, and sampling was performed at different time points: 0 hours (H0), 1 hour (H1), 3 hours (H3), 6 hours (H6), 12 hours (H12), 24 hours (H24), and 48 hours (H48).

To study the expression patterns of *PsTCP* genes in different tissues, samples from Light purple vexilla (LPV), Dark purple wing (DPW), and purple pod (PP) of the Yunwan 127 variety were collected. The remaining tissue samples were from the Zhongwan 6 variety. All plants were not subjected to significant artificial or natural stress before sampling, with irrigation managed by natural precipitation and supplemented by artificial irrigation when necessary. The samples were collected two days after flowering, on April 16, 2024, at 10:00 AM, with clear weather and a temperature of 21°C. The sampling location was at 36°42’12.45” N, 117°04’49.39” E. A total of 11 different tissue samples were collected with three biological replicates per tissue, each replicate representing one plant. The tissues collected were: white flower (WF), dark purple wing (DPW), light purple vexilla (LPV), normal stipule (NS), root ^®^, stem (S), fresh seed (FS), tendril leaf (T), imparipinnate leaf (IL), green pod (GP), and purple pod (PP). All collected samples were immediately frozen in liquid nitrogen and stored at −80°C for RNA extraction.

Total RNA was isolated from leaf tissues using TRIzol reagent, and the RNA quality and quantity were assessed using an Agilent 2100 Bioanalyzer. cDNA libraries were then constructed and high-throughput sequencing was performed on the DNBSEQ platform. To ensure data quality, all RNA sequencing data were filtered using Fastp (Chen et al., 2018) (default parameters). Clean reads were then aligned to the pea reference genome using HISAT2 (Kim et al., 2019) (v2.1.0, default parameters). Gene expression levels were quantified using the FeatureCounts tool (Liao et al., 2013), and Trimmed Mean of M-values (TMM) normalization was applied, with genes having expression levels lower than 1 TMM being filtered out.

### 3.8 WGCNA network construction and GO enrichment analysis

To construct a co-expression network under salt stress conditions in pea, we first imported transcriptome data (TMM values) from different NaCl treatments into R (v4.4.2) and used the WGCNA R package (Langfelder and Horvath, 2008) for network construction. No data filtering was applied, and a power value of 4 was chosen. Through WGCNA analysis, genes were grouped into different modules. Next, we extracted genes that co-expressed with the *PsTCP* gene family and used the ClusterGVis package (https://github.com/junjunlab/ClusterGVis) to plot the expression trend of these genes, with *k*-means clustering performed for analysis.

For GO enrichment analysis, we first annotated the pea protein sequences using ggnog-mapper v2 (Cantalapiedra et al., 2021) to obtain functional information. Then, we performed GO enrichment analysis on the genes co-expressed with the *PsTCP* family using the clusterProfiler R package (Wu et al., 2021). A significance level of P and Q values set at 0.05 was chosen for the analysis, which was categorized into biological process (BP), cellular component (CC), and molecular function (MF). The top 20 significantly enriched GO terms were selected for visualization to better elucidate the potential roles of these genes in the salt stress response of pea.

## 4 Discussion

### 4.1 Diversity and evolutionary conservation of *PsTCP* genes

The identification of 21 *PsTCP* genes in pea highlights the functional diversity within this gene family. These genes are unevenly distributed across seven chromosomes, with chromosome 4 harboring the highest number of family members (*PsTCP9* to *PsTCP13*) (**Supplementary Figure 1**, **Figure 4**). This uneven distribution suggests that gene duplication events may have driven functional diversification, enabling pea to adapt to various environmental stresses (Yang et al., 2020). The physicochemical properties of *PsTCP* proteins, including molecular weight (21.87–54.5 kDa) and isoelectric points (pI 5.88–9.51), indicate that these proteins may operate in diverse cellular environments, adapting to varying pH conditions (Sabri Dashti et al., 2012). Subcellular localization predictions reveal that most *PsTCP* proteins are localized to the nucleus, consistent with their roles as transcription factors, while *PsTCP9* is localized to the peroxisome, suggesting a potential role in ROS metabolism (Murakami et al., 2013).

Phylogenetic analysis classified *PsTCP* genes into Class I (PCF) and Class II (CYC/TB1 and CIN) subfamilies, mirroring their roles in growth and stress responses (**Figure 1**). The distribution of *PsTCP* proteins across subfamilies mirrors that in *A. thaliana*, highlighting shared evolutionary trajectories. This conservation suggests that TCP proteins play similar roles in regulating plant growth and development across species. The number of *TCP* genes in pea is fewer than in *A. thaliana*, likely due to differences in genome structure, gene duplication events, and selective pressures (Danisman, 2016). In contrast, the simpler genome of *A. thaliana* may have facilitated more extensive gene duplication (Ling et al., 2020).

### 4.2 Functional diversification and regulatory mechanisms

The conserved motifs identified in PsTCP proteins, such as motif 1 present across all family members, highlight their structural and functional stability (**Figure 3A**). Subfamily-specific motifs, such as motif 2 in the CIN subfamily, suggest distinct roles in specialized processes, such as flower development (Rath et al., 2022). The lack of introns in certain *PsTCP* genes, like *PsTCP17*, may be attributed to transposon activity, which could facilitate functional diversification (Venner et al., 2009). These structural features provide valuable insights into the potential functions, evolutionary history, and roles of *PsTCP* family members in plant growth, development, and stress responses.

*Cis*-regulatory element analysis further indicated that *PsTCP* genes harbor elements associated with growth, hormone signaling, and stress response (**Figure 5**). For example, *PsTCP7* contains the greatest number of functional elements (45), including 21 phytohormone-responsive elements, suggesting a high sensitivity to hormonal regulation (Ijaz et al., 2020). In contrast, *PsTCP20* harbors fewer hormone-responsive elements, which may imply reliance on alternative regulatory pathways. Notably, *PsTCP17* is characterized by the highest number of abiotic and biotic stresses elements (20), potentially enhancing its expression under stress conditions, thus supporting its role in stress adaptation.

### 4.3 Evolutionary relationships and functional diversification of *PsTCP* genes

Phylogenetic analysis reveals that *PsTCP* genes are classified into Class I (PCF) and Class II (CYC/TB1 and CIN) subfamilies, reflecting their roles in growth and stress responses (**Figure 1A–C**). The distribution of PsTCP proteins across subfamilies mirrors that in *A. thaliana*, highlighting shared evolutionary trajectories. This conservation suggests that TCP proteins play similar roles in regulating plant growth and development across species. The number of *TCP* genes in pea is fewer than in *A. thaliana*, likely due to differences in genome structure, gene duplication events, and selective pressures (Danisman, 2016). In contrast, the simpler genome of *A. thaliana* may have facilitated more extensive gene duplication (Ling et al., 2020).

Class I *TCP* genes, prevalent in *A. thaliana*, likely regulate fundamental transcriptional processes, while the CIN subfamily, more prominent in pea, suggests expanded roles. In *A. thaliana*, CIN genes regulate cell proliferation and differentiation during leaf development (Rath et al., 2022).Conserved motifs, such as motif 1 in all *PsTCP* proteins, underscore their structural and functional integrity, while subfamily-specific motifs (e.g., motif 2 in CIN) suggest specialized roles in processes like flower development (**Figure 3A**) (Rath et al., 2022). The absence of introns in some *PsTCP* genes (e.g., *PsTCP* and *PsTCP17*) may result from transposon activity, contributing to functional diversification (Venner et al., 2009).

### 4.4 Expression patterns and regulatory networks under salt stress

The expression patterns of *PsTCP* genes under salt stress reveal their dynamic roles in stress adaptation (**Figure 6**). In the experiments with different NaCl concentrations (0–120 mM), *PsTCP19* and *PsTCP8* were upregulated with increasing salt levels, suggesting their involvement in early stress responses, potentially through the regulation of ion homeostasis (Wang et al., 2022b). In contrast, *PsTCP4*, *PsTCP18*, *PsTCP20*, and *PsTCP12* were downregulated under high salt concentrations, indicating suppression of non-essential processes to conserve resources (Amirbakhtiar et al., 2021). Notably, *PsTCP17* showed significant upregulation under extreme salt stress (120 mM NaCl), highlighting its potential role in severe stress adaptation.

Time-course experiments with 100 mM NaCl treatment further elucidated the temporal dynamics of *PsTCP* gene expression (**Supplementary Figure 5**). *PsTCP5*, *PsTCP11*, and *PsTCP13* exhibited an initial increase followed by a decline, suggesting their involvement in early stress responses. Conversely, *PsTCP18* and *PsTCP7* showed a decrease followed by an increase, indicating potential roles in prolonged stress adaptation. The expression pattern of *PsTCP17* under prolonged salt stress mirrored its response to high salt concentrations, further supporting its critical role in extreme stress conditions.

WGCNA analysis identified *PsTCP8*, *PsTCP9*, *PsTCP12*, *PsTCP17*, and *PsTCP20* as hub genes within a co-expression network that regulates photosynthesis and metabolism under salt stress (**Figure 7B**). Genes within the blue module, including six *PsTCP* genes, were significantly enriched in pathways associated with photosynthesis and metabolism, such as “photosynthetic electron transport in photosystem I” and “chlorophyll metabolic process” (**Figure 7C**). The upregulation of these genes under low salt conditions (T30) suggests an enhancement of photosynthetic and metabolic processes to mitigate mild stress. Conversely, their downregulation under high salt conditions (T60 to T120) reflects the suppression of these essential processes under severe stress, likely due to the adverse effects of salinity on cellular functions (Parida et al., 2003; Sui et al., 2015; Ben Amor et al., 2020). The high connectivity of *PsTCP20* (degree = 2742) highlights its central role in coordinating the pea plant’s response to salt stress, while *PsTCP17* and *PsTCP12* may function synergistically to regulate stress adaptation pathways.

## 5 Conclusion

This study comprehensively identified and analyzed the *TCP* gene family in pea, revealing its crucial role in plant growth, development, and response to salt stress. A total of 21 *PsTCP* genes were identified, and these genes are unevenly distributed across seven chromosomes of the pea genome. Through a comprehensive analysis of their protein characteristics, phylogenetic relationships, three-dimensional structures, conserved motifs, gene structures, synteny, selective pressure, *cis*-regulatory elements in promoter regions, protein-protein interaction networks, and expression patterns under different tissues and salt stress conditions, we found significant diversity and functional differentiation within the *PsTCP* gene family. Under salt stress, *PsTCP* genes exhibited complex dynamic expression patterns, with *PsTCP17* significantly upregulated at high salt concentrations, potentially playing an important regulatory role under extreme salt stress conditions. *PsTCP20*, as a core hub gene in the co-expression network, showed high co-expression with genes related to photosynthesis and metabolism, indicating its key regulatory function in pea adaptation to salt stress. Furthermore, the differential expression of *PsTCP* genes across various tissues highlighted their diversified roles in plant growth and development. These findings not only enrich our understanding of the functions of the TCP gene family but also provide potential genetic targets and theoretical basis for the molecular improvement of salt-tolerant pea varieties and other crops. However, our current understanding of *PsTCP* gene functions is primarily based on bioinformatics predictions and expression analysis, and future studies should employ experimental approaches, such as gene knockout or overexpression, to directly validate their specific roles in pea.

## 6 Limitations of the study

Although this study provides important insights into the *PsTCP* gene family in pea, our current understanding of the functions of *PsTCP* genes primarily relies on bioinformatic predictions and expression analyses. The lack of direct functional validation methods, such as gene knockout or overexpression experiments, limits our deeper understanding of the specific functions and regulatory mechanisms of *PsTCP* genes.

## Data Availability

The datasets presented in this study can be found in online repositories. The names of the repository/repositories and accession number(s) can be found in the article/**Supplementary Material**.
