# Supplementary material for: Integrating genome and transcriptome-wide data to explore the expression dynamics of TCP genes in Pisum sativum under salt stress
Source: Front Plant Sci. 2025 May 1;16:1580890. doi: 10.3389/fpls.2025.1580890 (PMC12083428; doi:10.3389/fpls.2025.1580890)
Supplement: Supplementary file 1 [file SupplementaryFile1.docx]

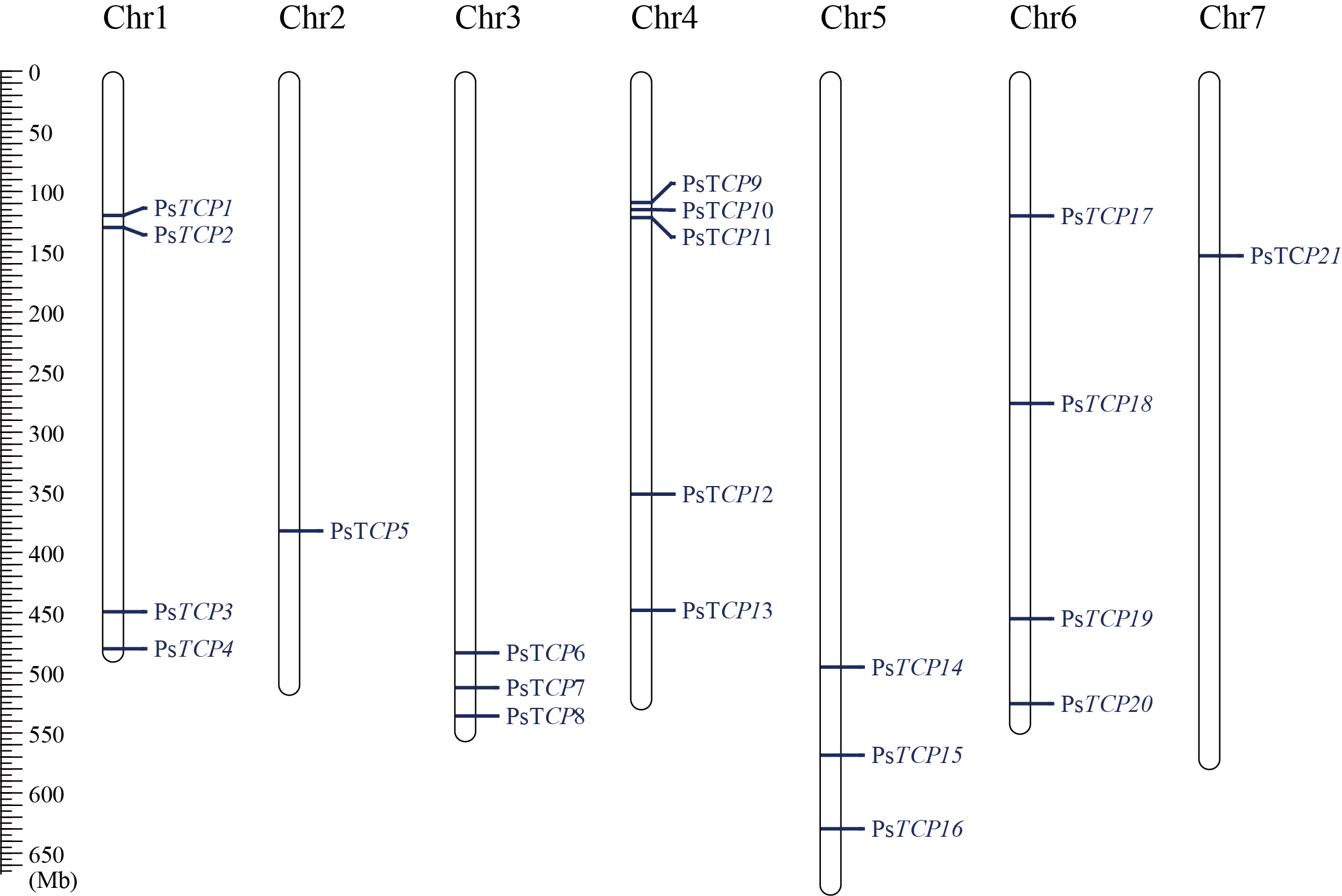


**Supplementary Fig. 1 Chromosomal localization analysis of the *PsTCP* gene family in pea.**


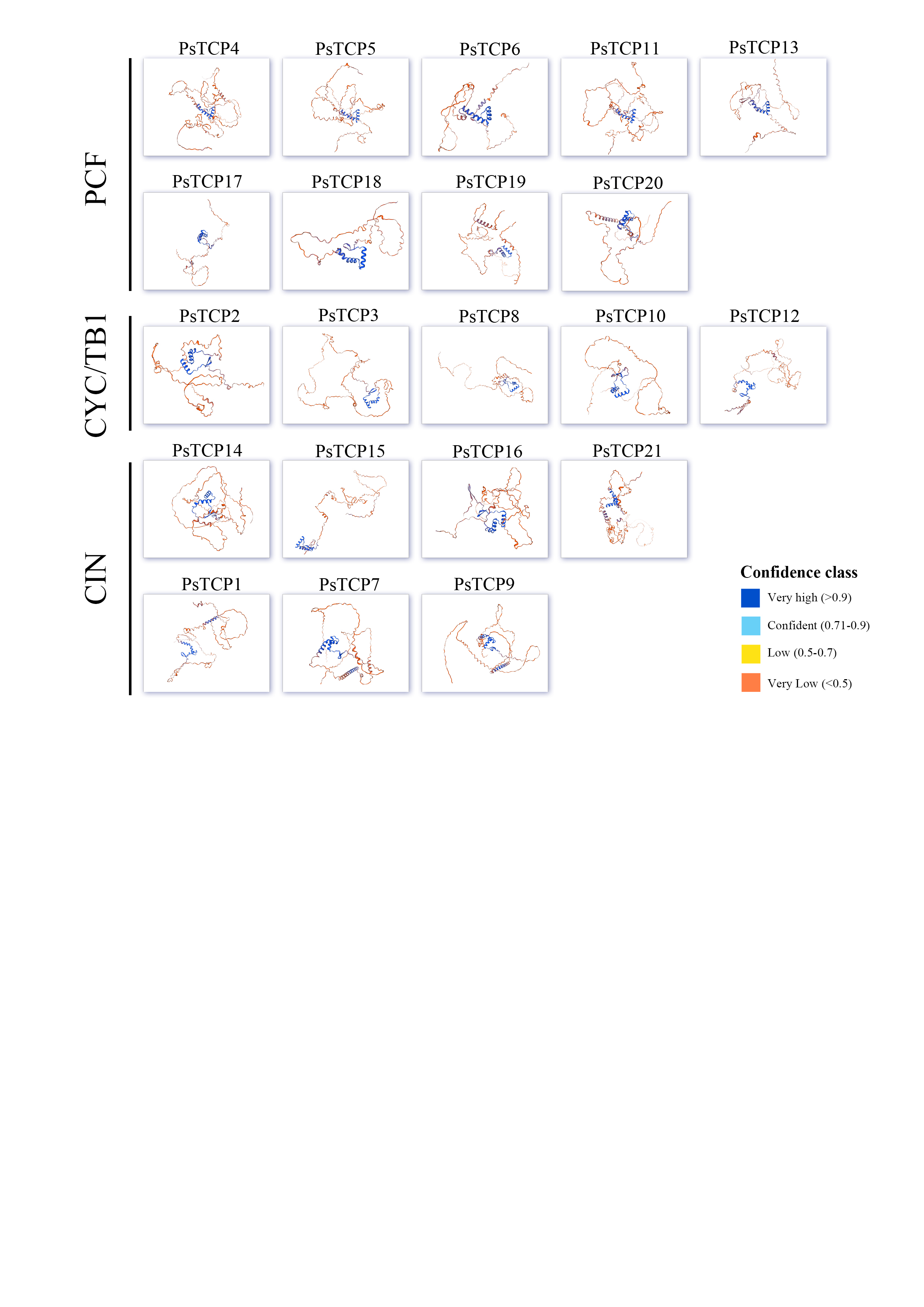


**Supplementary Fig. 2 Structural analysis of PsTCP proteins.** The left panel shows the classification of the three subfamilies, with different colors representing the confidence levels of the protein’s three-dimensional structure. The bottom-right corner provides a legend for the four confidence levels, with blue indicating the highest confidence and orange indicating the lowest confidence.


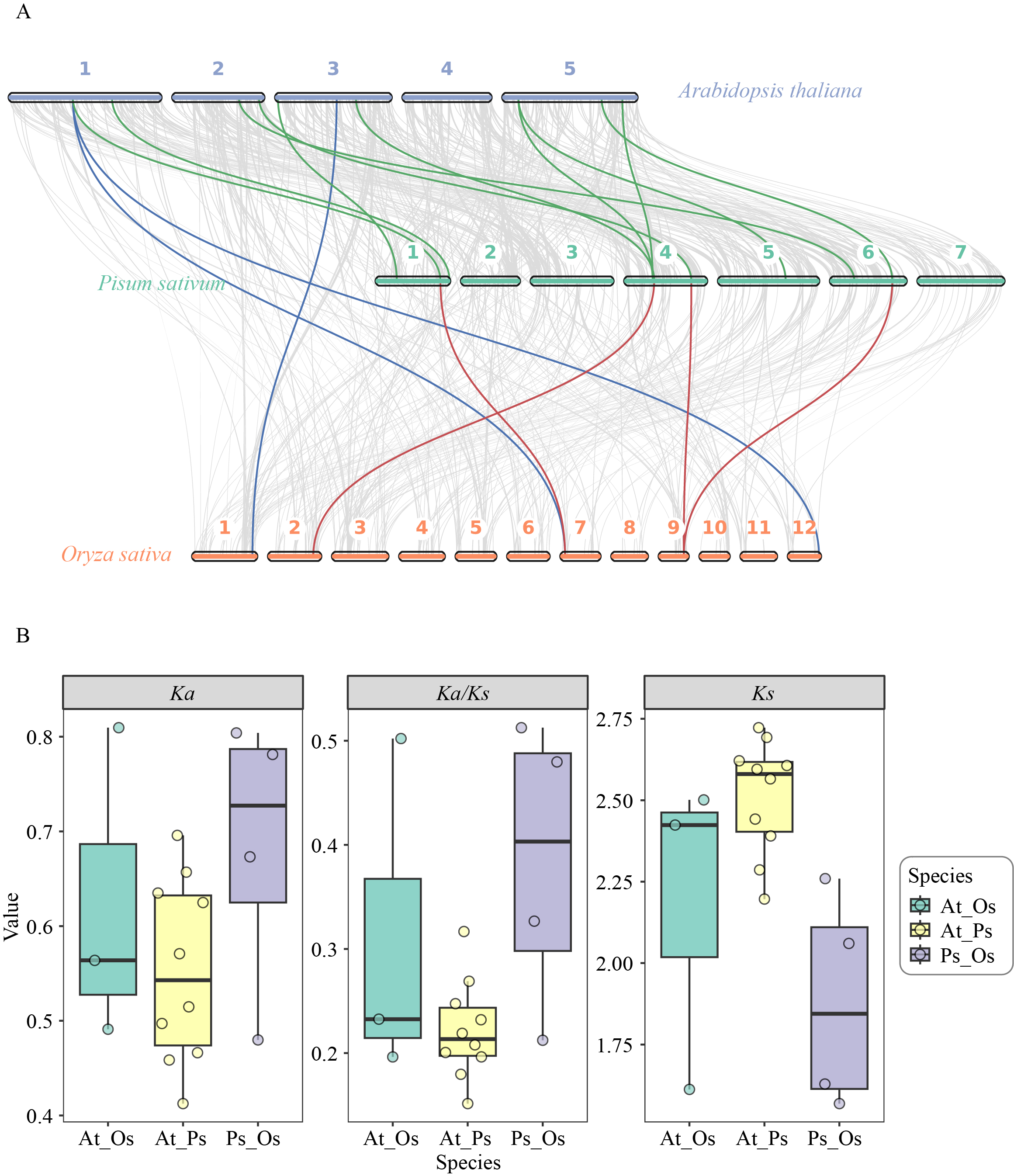


**Supplementary Fig. 3 Gene evolutionary analysis of pea, rice, and *A. thaliana*.** (**A**) Shows gene synteny between species. Blue, green, and orange rectangles represent the chromosomes of *A. thaliana*, pea, and rice, respectively. The numbers above the rectangles indicate the chromosome numbers. Gray lines represent syntenic regions between genomes, while green, blue, and red lines represent the orthologous relationships between *TCP* genes of *A. thaliana* and pea, *A. thaliana* and rice, and pea and rice, respectively. (**B**) Shows boxplots of nonsynonymous substitution rate (*Ka*), synonymous substitution ra te (*Ks*), and their ratio (*Ka/Ks*) between species.


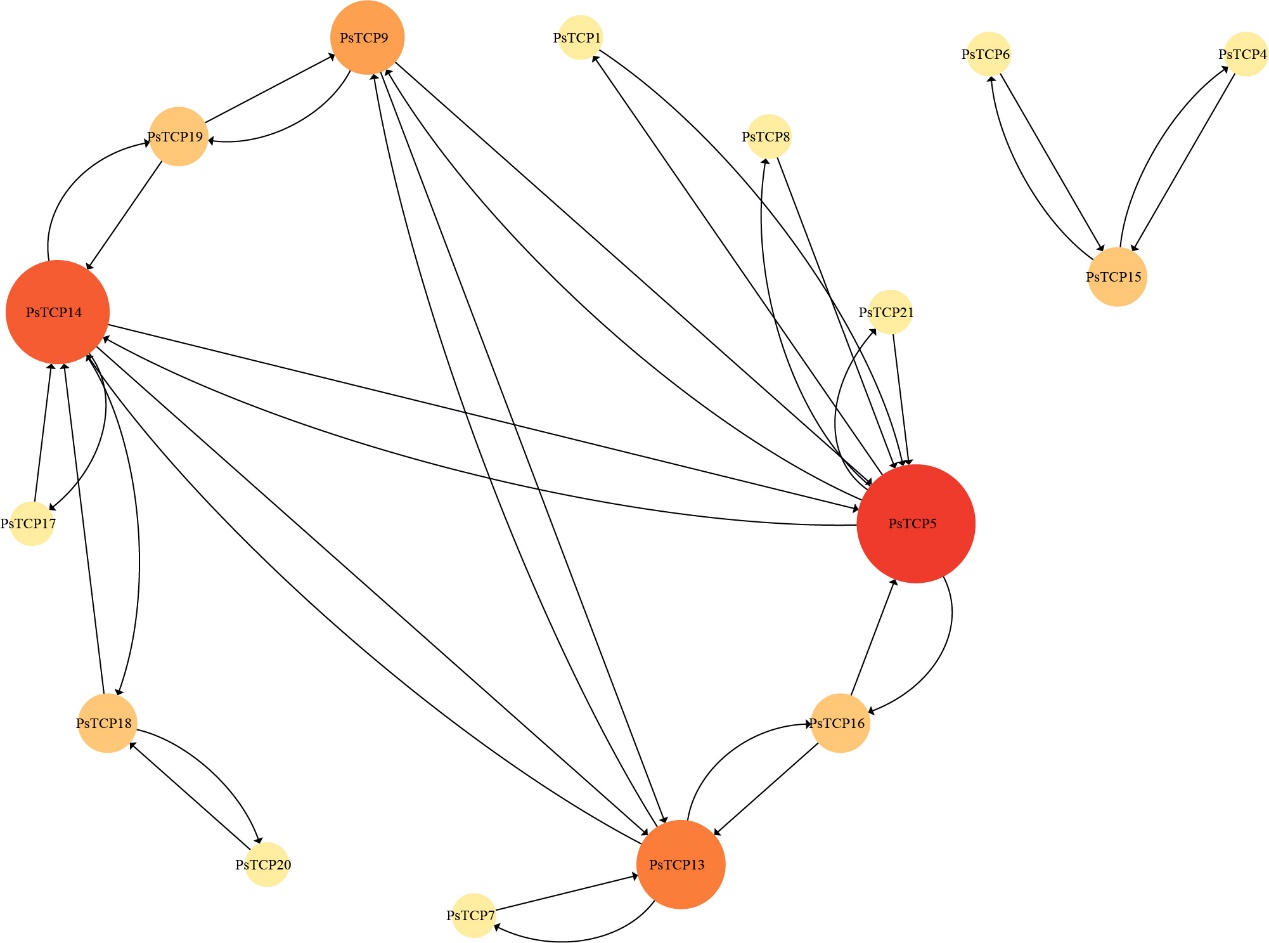


**Supplementary Fig. 4 Protein-protein interaction network.** The connectivity of genes is represented by the intensity of the color and the size of the circles. The darker the color and the larger the circle, the higher the gene's connectivity. Arrows indicate the regulatory relationships between genes, with arrows pointing to downstream genes.


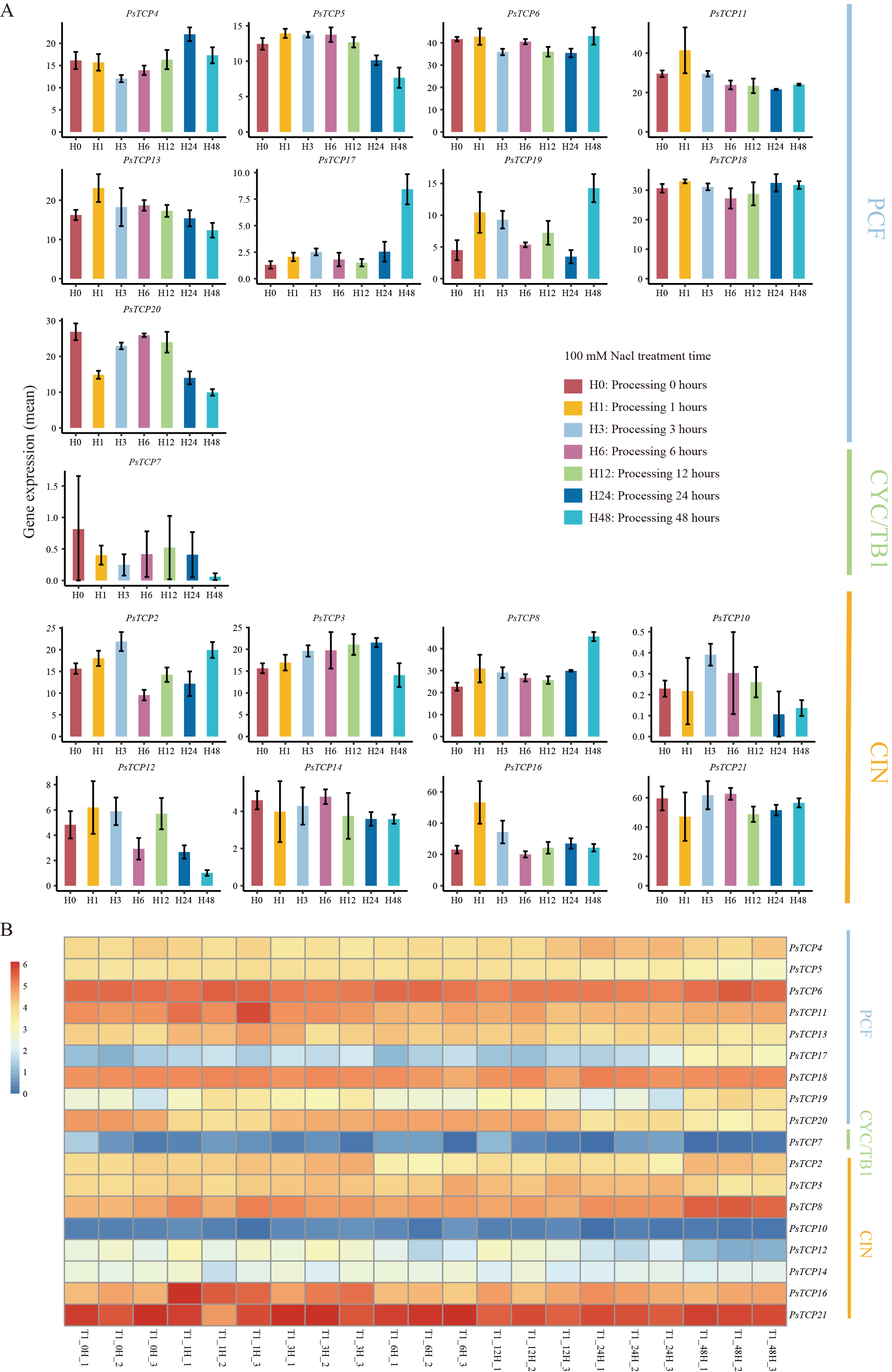


**Supplementary Fig. 5 Expression profiles of *PsTCP* genes under 100 mM NaCl stress at different time points.**

(**A**) Bar chart showing the expression levels. The x-axis represents the expression levels of different treatment groups, and the y-axis represents the time points of the treatments. Gene names are indicated above each subplot. The bar chart shows the average expression levels of the samples in each treatment group, with error bars representing the standard deviation (SD). (**B**) Heatmap of expression levels. Red indicates high expression, while blue indicates low expression. Gene names are labeled on the right side of the heatmap, with the treatment samples displayed below. The subfamily classification is shown on the right. Genes with expression levels below 1 have been filtered out.


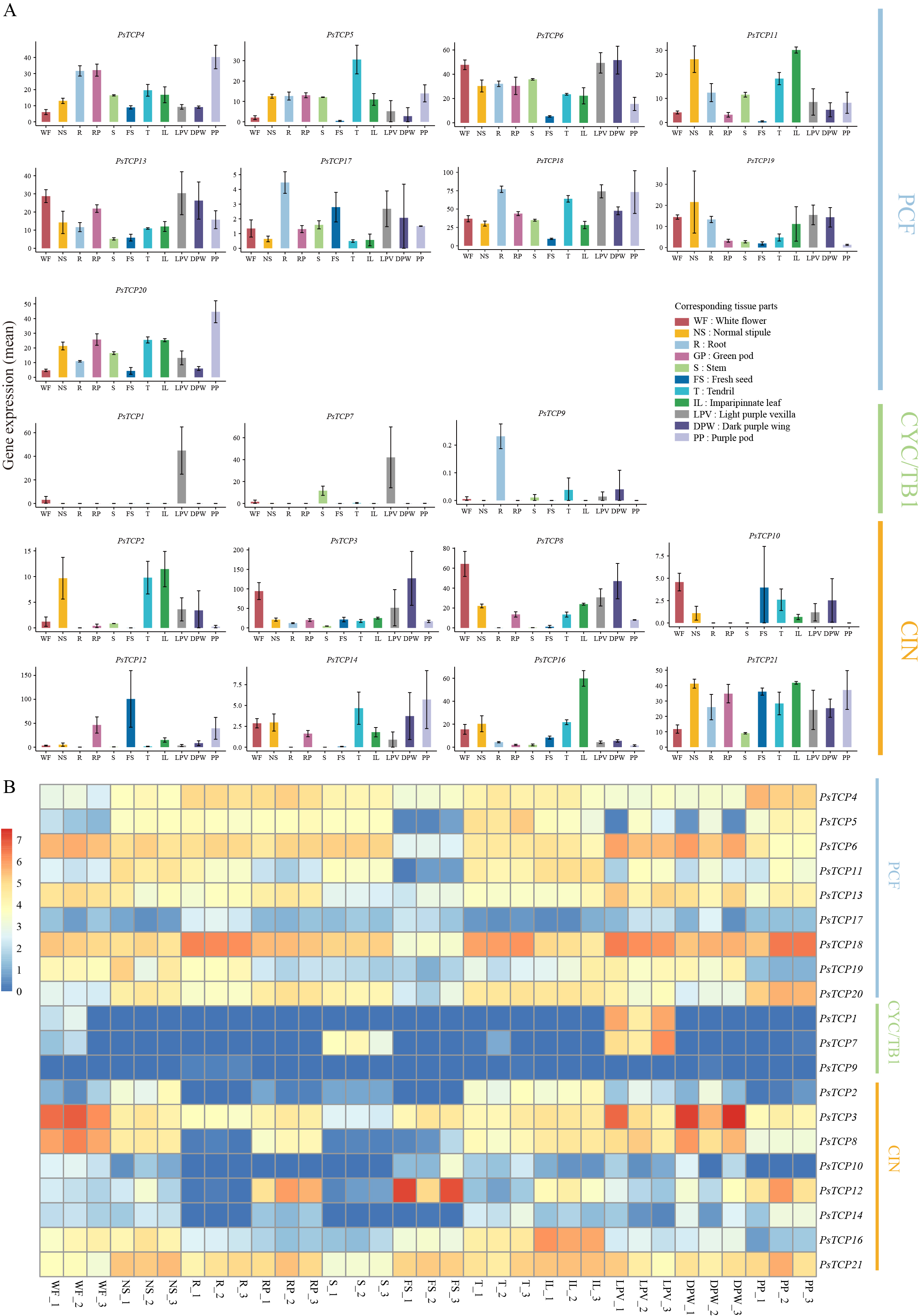


**Supplementary Fig. 6 Expression profiles of PsTCP genes in 11 different pea tissues.** (**A**) Bar chart of expression levels, with gene names labeled above each subplot. The x-axis represents expression levels, and the y-axis represents different tissue samples. The bar chart displays the average expression level of each tissue sample, with error bars representing standard deviation (SD). (**B**) Heatmap of expression levels, where red indicates high expression and blue indicates low expression. Gene names are displayed on the right side of the heatmap, and the tissue samples are labeled below. The right side of the figure indicates the classification of the three subfamilies. Genes with expression levels below 1 in all treatments have been filtered out.
